# Safety and Efficacy of Fully Covered Self-Expandable Metal Stents for Benign Upper Gastrointestinal Strictures Beyond the Esophagus

**DOI:** 10.7759/cureus.31439

**Published:** 2022-11-13

**Authors:** Navkiran K Randhawa, Ahamed Khalyfa, Mahnoor Khan, Nayab Ahsan, Mahnoor Inamullah, Kamran Ayub

**Affiliations:** 1 Internal Medicine, Franciscan Health, Olympia Fields, USA; 2 Gastroenterology, Icahn School of Medicine at Mount Sinai, New York, USA; 3 Gastroenterology, Southwest Gastroenterology, Oak Lawn, USA; 4 Gastroenterology, Silver Cross Hospital, New Lenox, USA

**Keywords:** gastric outlet obstruction, case series, fully covered self-expandable metal stents, upper endoscopy, benign strictures

## Abstract

Background

Stents utilized for pyloric, duodenal, or anastomotic malignant strictures are generally uncovered and are not retrievable. Taewoong Medical created a through-the-scope stent that is fully covered, retrievable, and can be placed beyond the esophagus for benign gastroduodenal strictures as an alternative to surgical approaches. The aim of this paper is to examine the safety and efficacy of short-term, fully covered, self-expanding metal stents (FC-SEMS) in refractory benign strictures of the pylorus, duodenum, and gastrojejunal anastomosis.

Methodology

This multicenter case series was conducted at four hospitals from January 2018 through December 2020. Patients presenting with benign strictures of the pylorus, duodenum, or gastrojejunal anastomosis were entered into the study. A therapeutic channel scope was utilized to place FC-SEMS to open strictures. The stents were removed a few weeks later. A four-point gastric outlet obstruction scoring system (GOOSS) was used to record improvement.

Results

Statistically significant improvement in GOOSS was found between the pre-procedure and the four-week follow-up.

Conclusions

Fully covered, retrievable metal stents appear safe and effective in the management of refractory benign pyloric, duodenal, and anastomotic strictures. This may provide a less invasive option in the management of these strictures compared to surgery.

## Introduction

Strictures of the upper gastrointestinal (GI) tract can be classified as either benign or malignant. The most common etiologies of benign upper GI strictures include peptic ulcer disease, chronic pancreatitis, extrinsic compression due to acute pancreatitis, post-surgical anastomotic strictures, or ischemic strictures. Common sites of benign upper GI strictures include the esophagus, the pylorus, the duodenum, and surgical anastomoses [1-3]. Palliative surgery and endoscopic stenting techniques are currently employed for the management of malignant strictures [4]. Traditionally, surgical procedures have been used as first-line treatment for benign and malignant strictures; however, after the advent of through-the-scope balloon dilators and endoscopic stenting, less invasive alternatives are now available, which have improved clinical outcomes [4-6]. Balloon dilation of these strictures has short-lived benefits in many cases as strictures tend to recur within a few days [6]. Palliative surgery and endoscopic stenting techniques are currently employed for the management of malignant strictures [4]. Fully covered, self-expanding metal stents (FC-SEMS) have increasingly become the standard of care for the palliative management of malignant upper GI strictures and have also been successfully used for palliative treatment in antro-pyloric and duodenal strictures [7]. The purpose of stenting is to reestablish the patency of the GI lumen to maintain adequate nutritional intake and improve quality of life [8]. The stents consist of interconnecting rows of metal which are assembled into a tube-like structure and are manufactured as uncovered, partially covered, and fully covered products [9,10]. Uncovered stents develop tissue ingrowth through the mesh and become embedded permanently. Therefore, these stents are for permanent use in malignant strictures and are not indicated in benign strictures [10]. FC-SEMS have a membrane to prevent tissue ingrowth; however, they have a much greater tendency to migrate as these stents do not get embedded into the tissue [10-14]. This study was designed to evaluate the safety and short-term efficacy of FC-SEMS for refractory benign strictures involving the pylorus, duodenum, and gastrojejunal anastomosis. In this study, the stents were sutured to prevent potential migration. These sutures can be cut and removed after a few weeks to provide satisfactory dilation of refractory benign strictures with long-term benefits.

## Materials and methods

Ethics approval was obtained from the Oak Lawn Ethics Committee of Illinois Endoscopy Society to conduct the study. Consent was obtained for each patient included in the study. Patients with significant luminal narrowing exhibiting symptoms of upper GI obstruction such as nausea, vomiting, upper abdominal pain and/or discomfort, and bloating were included in the study. Luminal narrowing was confirmed via upper GI series and/or esophagogastroduodenoscopy (EGD). This multicenter study was conducted at four different hospitals (Silver Cross Hospital, IL, USA; Amita Saint Joseph, IL, USA; Advocate Christ Medical Center, IL, USA; Good Samaritan Hospital, IL, USA). All procedures were performed by a single, advanced endoscopist. The inclusion criteria included the presence of symptomatic upper GI tract strictures beyond the esophagus that were refractory to balloon dilation. The exclusion criteria included any contraindication to upper endoscopy or stent placement or inability to obtain consent. FC-SEMS with the scope deployment capability produced by Taewoong Corporation were used in all patients for treatment of stated symptoms. The stents were placed under endoscopic and fluoroscopic guidance. The FC-SEMS were inserted through the therapeutic channel of the endoscope and were deployed across the site of stricture. The size of the stents used was either 18 mm or 20 mm in diameter (Table 1).

**Table 1:** Size of stents used.

Size of stent	Number of patients using stated stent
18 mm × 10 cm	3
18 mm × 6 cm	13
20 mm × 6 cm	9
20 mm × 10 cm	3

These stents were then sutured in place. Patients were followed and monitored for symptomatic improvement and complications. Oral intake was permitted on post-procedure day one, starting with a full liquid diet and gradually progressing to a soft diet if no complications occurred. Patients were first classified under a four-point gastric outlet obstruction scoring system (GOOSS) pre-procedure (Table 2).

**Table 2:** Gastric outlet obstruction scoring system (GOOSS).

GOOSS points	Symptoms
0	No oral intake
1	Liquid diet
2	Soft diet
3	Low residue or regular diet

The patients were re-evaluated at week one and week four post-procedure and were similarly scored based on GOOSS. A minimum of one-point improvement in GOOSS was considered significant. To remove the stents follow-up EGD was performed two to three weeks after FC-SEMS placement in pyloric and duodenal strictures and after four to six weeks in gastric bypass and Whipple jejunostomy anastomosis. FC-SEMS were removed using biopsy forceps or Rat Tooth Forceps during the follow-up EGD. The sutures were cut using Enzisor Endoscopic Scissors or loop cutters. Long-term follow-up was performed in all patients with a minimum follow-up duration of four months to a maximum of 24 months. Repeat EGD with FC-SEMS placement was performed in case of recurrent obstructive symptoms and restenosis on long-term follow-up. A chi-square test of independence was used to determine the significance of clinical improvement based on GOOSS pre- and post-procedure.

## Results

The study involved 28 symptomatic patients with benign upper GI strictures. These patients had significant luminal narrowing combined with symptoms of upper gastric outlet obstructions which included nausea, vomiting, upper abdominal pain and/or discomfort, and bloating. In total, 13 patients had strictures in the duodenum, six in the pylorus, six in the gastric bypass anastomosis, and three with Whipple gastrojejunostomy and efferent limb obstruction. Luminal narrowing was confirmed via upper GI series and/or EGD. Eight patients had their duodenal stents receive one suture for fixation. The remaining patients received two or three sutures for the fixation of their respective stents. Suturing was performed using the Apollo Overstitch Device (Apollo, USA).

Statistical results

Statistical analysis was performed on pre-procedure and post-procedure data on the GOOSS score. Pre-procedure, one-week post-procedure, and four-weeks post-procedure GOOSS scores were documented (Table 3). Chi-square analyses revealed significant changes from GOOSS 1 to GOOSS 2 in the interim from the pre-procedure and the first week (χ^2^ = 13.89; p < 0.001; 1 df). A significant change was also found between the pre-procedure and the four-week follow-up from GOOSS 1 to GOOSS 2 (χ^2^ = 21.90, p < 0.001, 1 df). When looking for overall improvement in GOOSS score, significance was only found between pre-procedure and one-week follow-up (χ^2^ = 6.72, p < 0.05, 1 df).

**Table 3:** Respective numbers of gastric outlet obstruction scoring system (GOOSS) of patients pre-procedure, one-week post-procedure, and four-week post-procedure.

GOOSS	Pre-procedure	One-week post-procedure	Four-week post-procedure
0	6	0	0
1	18	3	0
2	4	12	13
3	0	13	15

The data suggests that the stent procedures are significantly effective in cases with GOOSS scores greater than zero. Patients with a GOOSS score of 1 were seen to improve to a GOOSS score of 2 at both the one-week and four-week follow-up. Improvement in higher score patients was not evident in the pre-procedure to one-week post-procedure GOOSS scores, but it was significant from pre-procedure to four-week follow-up scoring.

Complications

FC-SEMS migration occurred in eight patients. Three patients had distal migration while five had proximal migration into the stomach. Among patients who experienced distal migration, two had a spontaneous passage of FC-SEMS from the alimentary tract and one had the stent removed by double-balloon enteroscopy. Five patients had proximal migration of stents into the stomach, and all patients had the sutures intact and the stents seemed to have retracted into the stomach. These stents were removed after cutting the sutures using Enzisor Endoscopic Scissors or loop cutters. Subsequently, biopsy forceps or Rat Tooth forceps were used to pull the proximal end of the stent into the distal attachment. Migrations were observed in patients who received one suture for fixation of the duodenal stent. No distal migration of the duodenal stents was observed in patients who received two or three stitches for fixation. Two patients had a recurrence of obstructive symptoms; one patient was noted to have antro-pyloric stenosis (six months post-procedure) while the other one had gastric bypass anastomosis (12 months post-procedure). Both patients were treated with a repeat EGD with FC-SEMS replacement. Post-procedure pain was noted in eight patients and this required narcotic analgesics for approximately four days. Other complications, including perforation, tissue ingrowth/overgrowth, and hemorrhage, did not occur in any patient.

## Discussion

Stents have been used to maintain luminal patency and widen a stenosed lumen. Originally, stents were composed of hard plastic and were typically utilized for obstructive esophageal malignancies. The stents utilized today are composed of metal and are either made with stainless steel or nitinol. Nitinol mesh consists of a nickel-titanium alloy allowing it to be soft and flexible. The wire ends are smoother resulting in a reduced risk of tissue overgrowth. These metal stents can be uncovered, partially covered, or fully covered. Only FC-SEMS were utilized in the study. This prospective study demonstrates a significant improvement in gastric outlet obstructive symptoms up to four weeks post-FC-SEMS placement in all patients. The only immediate complication noted in this study was the migration of the stents in eight patients. Long-term follow-ups did document the recurrence of obstructive symptoms in two patients; however, they were treated by repeat EGD with FC-SEMS replacement without complications. Thus, FC-SEMS placement for benign, refractory upper GI strictures appears to be a safe and effective treatment.

A therapeutic option, endoscopic ultrasound-guided gastrojejunostomy (EUS-GJ), involves the identification of the jejunum directly from inside of the stomach with subsequent placement of a lumen-apposing metal stent. Target identification, as well as proper stent placement, are challenging steps for the EUS-GJ technique, and this technique is only available at a few centers. Furthermore, patients with pyloric obstruction associated with chronic pancreatitis are especially resistant to endoscopic balloon dilation [5].

Another treatment modality is surgical gastrojejunostomy which can typically be performed in patients with pyloric or duodenal obstruction but not in patients with gastric bypass anastomotic strictures or post-Whipple strictures. Traditionally, gastroduodenal obstruction has been treated by various surgical gastrojejunostomy techniques. Although it has been an effective treatment, these procedures result in operative morbidity requiring hospitalization [4,15]. In our prospective study, all procedures were performed as outpatients, and patients were able to tolerate a soft diet by day one post-procedure. None of the complications of surgical gastrojejunostomy, including hemorrhage, delayed gastric emptying, wound infections and cellulitis, fever, confusion, renal failure, myocardial infarction, and deep vein thrombosis, were observed in our patients [4,15]. Fully covered stents can migrate from the site of endoscopic placement [9,16]. Various anti-migration techniques have been attempted, including endoscopic clipping. This technique has been effective in preventing the migration of covered stents [17]. We felt that suturing the stent using the Endostich device provides better anchoring of the stent with less chance of subsequent migration. In this prospective study, the initial eight patients had their stents secured by a single suture. After the migration was observed in several of these patients, the rest of the patients received two to three sutures at the discretion of the endoscopist for fixation of the stents. With this strategy, no distal migration was observed. When compared to gastrojejunostomy for the treatment of gastric outlet obstruction, endoscopic stenting with FC-SEMS is an elective procedure thus avoiding hospital stay, shorter time to tolerating oral intake, reducing healthcare costs, and a lower rate of complications [18]. Additionally, in a systematic literature review involving 1,343 patients, Jeurknink et al. concluded a higher clinical success with endoscopic stenting in terms of relief of symptoms with improved oral intake, when compared to gastrojejunostomy [19-21]. In our prospective study, symptomatic relief was observed immediately after FC-SEMS placement with all patients tolerating a soft diet by the end of the first post-procedure day.

The limitations of our study include a relatively small sample size and a lack of a comparison with surgical gastrojejunostomy or EUS-GJ counterparts. Future studies utilizing a larger sample size and randomized comparison to surgical gastrojejunostomy and/or EUS-GJ will be required to further validate this technique.

## Conclusions

In patients with refractory, benign strictures of the pylorus, duodenum, gastric bypass, and Whipple gastrojejunostomy anastomosis, the placement of FC-SEMS with two to three sutures appears to be a safe and effective modality as it resolved obstructive symptoms and treated benign strictures with minimal risk of complications. Additionally, applying two to three sutures to secure the stents appears to reduce the risk of migration when compared to using one suture.
